# Mapping livestock movements in Sahelian Africa

**DOI:** 10.1038/s41598-020-65132-8

**Published:** 2020-05-20

**Authors:** Camille Jahel, Maxime Lenormand, Ismaila Seck, Andrea Apolloni, Ibra Toure, Coumba Faye, Baba Sall, Mbargou Lo, Cecile Squarzoni Diaw, Renaud Lancelot, Caroline Coste

**Affiliations:** 1 CIRAD, UMR TETIS, Montpellier, France; 2 ISRA, BAME, rue des Hydrocarbure, Dakar, Senegal; 3 PPZS, Pastoral Systems and Dry Lands, Dakar, Senegal; 4 TETIS, Univ Montpellier, AgroParisTech, Cirad, CNRS, INRAE, Montpellier, France; 5 DSV, Dakar, Senegal; 6 FAO-ECTAD Regional Office, Accra, Ghana; 7 CIRAD, UMR ASTRE, Campus International de Baillarguet, 34398 Montpellier, France; 8 ASTRE, Univ Montpellier, CIRAD, INRAE, 34398 Montpellier, France; 9 ISRA, LNERV, rue fond de terre, Dakar, Senegal; 10 CIRAD, UMR SELMET, Campus International de Baillarguet, 34398 Montpellier, France; 11 CIRAD, UMR System, 2 place Viala, 34060 Montpellier, France; 12 French Agricultural Research for Development (CIRAD), French National Institute for Agricultural Research (INRA), Montpellier, France, UMR ASTRE, F-34398 Montpellier, France; 13 French Agricultural Research for Development (CIRAD), French National Institute for Agricultural Research (INRA), F-97490 Sainte Clotilde, Reunion Island France

**Keywords:** Infectious diseases, Applied mathematics, Applied physics

## Abstract

In the dominant livestock systems of Sahelian countries herds have to move across territories. Their mobility is often a source of conflict with farmers in the areas crossed, and helps spread diseases such as Rift Valley Fever. Knowledge of the routes followed by herds is therefore core to guiding the implementation of preventive and control measures for transboundary animal diseases, land use planning and conflict management. However, the lack of quantitative data on livestock movements, together with the high temporal and spatial variability of herd movements, has so far hampered the production of fine resolution maps of animal movements. This paper proposes a general framework for mapping potential paths for livestock movements and identifying areas of high animal passage potential for those movements. The method consists in combining the information contained in livestock mobility networks with landscape connectivity, based on different mobility conductance layers. We illustrate our approach with a livestock mobility network in Senegal and Mauritania in the 2014 dry and wet seasons.

## Introduction

Every year in West Africa, millions of animals move from the Sahelian semi-arid regions, where they were bred, towards southern regions looking for better grazing areas, or to be sold on consumption markets^1–3^. These movements often cause conflicts with farmers, especially during the wet growing season, when animals can invade cultivated plots^4–6^. Livestock trade mobility is also a key driver in spreading animal diseases. Indeed, on their way, livestock may cross areas with a high prevalence of mosquitos (lowlands, wetlands), which are vectors of diseases. The contact between animals when herds meet each other, is also conducive to disease transmission. Mapping movement patterns is thus essential for improving many aspects of livestock management at regional and national level, such as the management of natural resources, the positioning of borehole installations, the reduction of conflicts, and the control of animal diseases. However, the intrinsic complexity of livestock mobility paths makes it extremely tricky to map them.

One way of mapping livestock spatial distribution consists in working from a census or estimation of the number of animals at different resolutions. Some recent work improved the mapping of static livestock distribution by disaggregating census counts of animals, but provided no information about their actual movements. For instance, Tran *et al*.^7^ disaggregated census data taken at administrative level to produce risk maps for Rift Valley fever and Napp *et al*.^8^ used buffer areas to disaggregate their static data. Fournié *et al*.^9^ used densities derived from human demographic data, aggregated at village level, to study the transmission of Peste des Petits Ruminants. However, these approaches are limited to a static vision and do not enable animal movements to be explicitly taken into account.

We recently witnessed the emergence of network-based approaches to study livestock movements^10–12^. Such methods have been tested in many African countries^13–18^. It consists in describing livestock movements as a directed and weighted spatial network, where nodes represent villages, markets or premises and each link between two nodes represents at least one animal moving from one site to another. The weight of a link is equal to the total number of animals exchanged. In some ideal cases, the spatial pathway of the links is known, thanks to GPS tracking of animals^19^, but in Sahelian areas such data are rarely available and have only been tested on a few cattle^20,21^. Thus, the majority of livestock network analysis studies do not explicitly spatialize animal pathways between two nodes; the flows of the graph only provide information about the direction, distance and volume of movements.

Here we propose a way of mapping livestock movements that combines the information contained in livestock mobility networks with a landscape connectivity-based approach. The method consists in producing a conductance map representing the ease of livestock movements, to be linked with the mobility network in order to produce a map of potential paths. We illustrate our approach with a livestock mobility network in Mauritania and Senegal during the 2014 dry and wet seasons. The next section presents the proposed framework and the data used to illustrate our approach. The results are then presented, demonstrating the capacity and robustness of our approach in identifying potential paths for livestock movements in Sahelian Africa. Lastly, we discuss the advantages and limitations of our approach.

## Material and methods

### Study area

Our study area encompasses Senegal and Mauritania, where a recent report estimated the total number of cattle to be between 2 and 3 million^22^. In Mauritania, rangelands are predominant, with agricultural areas being limited to irrigable or flooded areas along the Senegal River and in oases. In Senegal, livestock farming is mostly located in Ferlo, a region of 70,000 km^2^ in the North east of the country, where climatic conditions do not allow the development of agricultural activity. A large share of the cattle spend the wet season in this rangeland area of Mauritania and northern Senegal, then moves towards the markets, or towards the crop residues of the central and southern regions, especially in the groundnut basin of Senegal. This animal trade mobility network between Mauritania and Senegal involves up to 1.9 million bovines^16^. Fewer than 20% of these animals are conveyed by vehicles, mostly commercial requests for religious feasts, with the rest traveling on foot, over a distance of one to three hundred kilometers^16^. Conveyance on foot enables the cattle to benefit from the pastures and crop residues of southern regions in order to continue fattening along the way. Animals traveling on foot often cross large areas before arriving at their final destination. At the border, large cattle herds will cross at official passage points, but the majority of herders use non-official points to avoid paying taxes, or because they are more accessible^16^, increasing the difficulty of mapping their paths.

### Livestock mobility network

**Figure 1:**
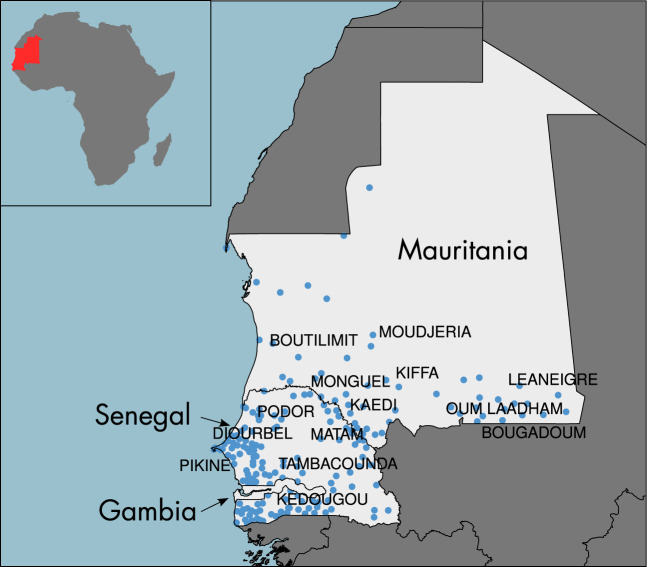
Positions of the nodes of the livestock mobility network. Each point corresponds to a market represented by a node in the livestock mobility network. The inset shows the location of the studied area in Africa.

Livestock mobility data are collected by field Veterinarian Services in Senegal, Gambia and Mauritania. In those countries, a certificate system based on sanitary movement permits (Sanitary Laissez-Passer or LPS) has been set up to keep track of animal mobility and map the main axes of movements in the area. Every time herders move their herds towards markets, or to other grazing area, a certificate is issued declaring, among other things, the date, the location of origin, the location of destination, the species and number of head, and the means of transportation. In this article, we consider only information relative to cattle movements, on foot, in 2014. We aggregated our data on a timescale of one month, providing a representation of the mobility dynamics over the year. This mobility information is represented by a weighted and directed livestock mobility network where the nodes correspond to the origin and destination locations (Fig. 1), and a directed link exists between two nodes if at least one animal is exchanged from one location to another. A link is characterized by the number of head exchanged (volume) and the month of occurrence. We distinguished between the characteristics of the network during the wet season (June to October) and the dry season (November to May).

We used several centrality metrics to analyze the weighted and directed livestock mobility network described above. We focused on five measures, the in- and out-degree (total number of links ingoing to a node or outgoing from a node, respectively), the in- and out-strength (total number of animals ingoing to a node or outgoing from a node, respectively), and the betweenness. The betweenness of a node is proportional to the number of shortest paths (weighted by the distance) going through this node.

### Mapping potential paths for livestock movements

**Figure 2:**
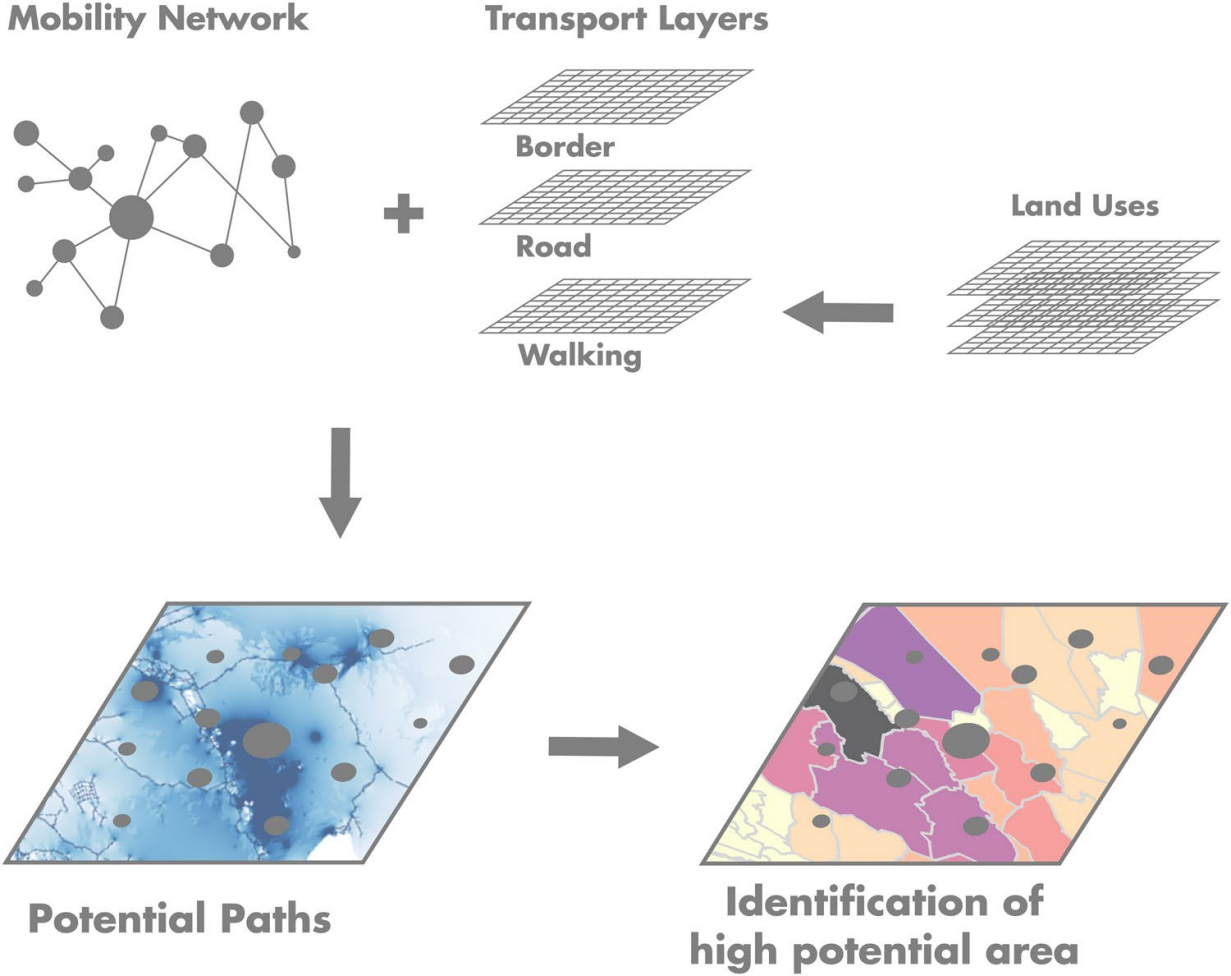
Methodology used to map potential paths for livestock movements and identify areas with a high potential for livestock movements based on mobility network and land use information.

As depicted in Fig. 2 the main purpose of the proposed methodology is to combine the information contained in the livestock mobility network described above and land use information to map the potential paths for livestock movements at high spatial resolutions. This section describes in detail the methods used to build the conductance map and to assign a potential route between every pair of nodes of our livestock mobility network based on this conductance map. Hereinafter referred to as landscape connectivity approach.

#### Conductance map

We used land-use/land-cover information and transportation features in Senegal and Mauritania to develop conductance maps represented as rasters at 500-meter resolution. Conductance is the reciprocal of resistance and therefore represents a greater ease of livestock movements. We assigned to each pixel of the conductance map a value according to its livestock movement propensity, ranging from 0 (low conductance/high resistance) to 1 (high conductance/low resistance). It is important to note that a pixel with no value (see Table 1) means that it is not possible to go through this pixel. We then applied an iterative process based on three different levels of information described below. Each geographical layer was rasterized to the same extent with a pixel dimension of 500 × 500 m^2^.

**Figure 3:**
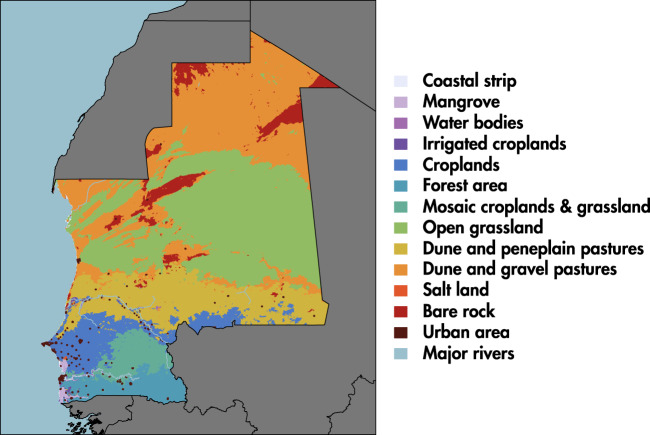
Land use map.
- The main road network in Senegal, Gambia and Mauritania downloaded from OpenStreetMap (data available online at https://www.openstreetmap.org, last accessed 18/02/2020). A map of the road network is available in Supplementary Information (Fig. S1).
- The administrative border line between Senegal and Mauritania comes from the GADM web platform (data available online at https://gadm.org/, last accessed 18/02/2020). The border crossing points (red points in Fig. S1 in Supplementary Information) were given by an expert from a Senegalese’s institute specialist in cattle mobility.

- A walking layer based on land use and land cover information provided by the FAO (data available online at http://www.fao.org, last accessed 14/06/2019). The original classification has been aggregated in 14 land-use types available in Fig. 3 and Table 1.

**Table 1:** Land use weights according to the season.

Type	Dry season	Wet season
Coastal strip	0.5	0.5
Mangrove	0.25	0.25
Water bodies	0.5	0.25
Irrigated croplands	—	—
Croplands	1	0.125
Forest area	0.5	0.5
Mosaic croplands & grassland	1	0.5
Open grassland	1	1
Dune and peneplain pastures	0.875	0.375
Dune and gravel pastures	1	0.875
Salt land	1	0.75
Bare rock	0.75	0.75
Urban area	0.125	0.125
Major rivers	—	—

The weights represent the conductance from 0 (low conductance/high resistance) to 1 (high conductance/low resistance). The symbol’-’ (no value) indicates that no movement is possible.

The bottom level of information regarding livestock movements is called the walking layer *W*. On this layer, conductance is based on landscape features and changes according to the season. We relied on expert knowledge to assign a conductance weight to each type of land use (Table 1). To do so, we conducted four individual interviews with experts, asking them to rank and then estimate the conductance value of different types of land use according to their knowledge of breeder mobility strategies. We analyzed the results with a fifth expert to choose the final values. The experts were researchers from French or Senegalese institutes and were specialists in cattle mobility, or members of Senegalese governmental institutions in the livestock sector.

The second level of information is represented by the main road network in Senegal and Mauritania. It is combined with the walking layer assigning the conductance value 1 (high conductance/low resistance) to any pixels of *W* crossed by a road to obtain a new layer *R*. Note that the influence of *W* on *R* can be adjusted with the parameter *δ*_*W*_ ∈ [0, 1]. More formally, the value *R*_*i*_ of a pixel *i* according to the walking layer *W* and *δ*_*W*_, is defined as follows,

1 $${R}_{i}=(\begin{array}{cc}1 & {\rm{if}}\,{\rm{a}}\,{\rm{road}}\,{\rm{cross}}\,i\\ {\delta }_{W}{W}_{i} & {\rm{otherwise}}\end{array}$$

Finally, the last level of information is given by the administrative border line. To adjust the permeability of the border line to pixels that are not border crossing points, we introduced the parameter *δ*_*R*_ ∈ [0, 1]. The value *C*_*i*_ of a pixel *i* on conductance map *C* according to *R* and *δ*_*R*_ is given by:

2 $${C}_{i}=(\begin{array}{cc}{\delta }_{R}{R}_{i} & {\rm{if}}\,i\,{\rm{is}}\,{\rm{not}}\,{\rm{a}}\,{\rm{border}}\,{\rm{crossing}}\,{\rm{point}}\\ {R}_{i} & {\rm{otherwise}}\end{array}$$

#### Livestock movement modeling

The last step consisted in assigning a potential route between every pair of nodes of our livestock mobility network using the conductance map described in the previous section. To do so, we conducted a connectivity analysis based on concepts from electronic circuit theory^23^ using Circuitscape software (v4) (https://pypi.org/project/Circuitscape/, last accessed 18/02/2020). This approach has been widely used in wildlife corridor design^24,25^, movement ecology^26,27^, and epidemiology^28^.

For each pair of locations, represented by two pixels on the conductance map, Circuitscape computes a map of the total movement resistance accumulated from the origin and destination based on the electronic circuit theory applied on the conductance map^23^. This map informs us about the potential for each pixel to be crossed during a livestock movement from the market of origin to the market of destination. We then normalized the map by its highest pixel value.

Then, we multiplied each normalized connectivity map by the ratio of animals concerned (i.e. number of animals moving from the origin to the destination divided by the total number of animals). We finally summed all the maps. We obtained a final map of the potential path for livestock, presented in the next section, where the highest values indicate the highest potential for livestock movements.

### Identification of high potential areas

In animal health programs, land-use planning, or management of conflicts between farmers and herders, it is essential to be able to prioritize intervention zones. To do so, we need to spatially aggregate the information contained in the maps of potential paths for livestock movements in order to identify high potential areas. In this study, we spatially aggregated the maps of potential paths at regional level for Senegal, Gambia and Mauritania, using data downloaded from the GADM web platform (https://gadm.org/index.html, last accessed 18/02/2020). We thus obtained a distribution of values informing us about the level of activity within each administrative unit based on the potential for each 500 × 500 m^2^ pixel to be crossed during a livestock movement. To facilitate the interpretation, the level of activity has been normalized by its maximum value and used to rank the different administrative units. We can also compute the level of normalized activity in each administrative unit based on the information provided by the livestock mobility network to compare the different approaches. In this case the activity is based on the total number of animals transiting in the administrative unit (sum of the in- and out-strength of the nodes located in the administrative unit).

To compare the different methods (landscape connectivity or network approaches) or the results obtained for different seasons, the distance between distributions of normalized activities (i.e rankings) can be assessed with the Kendall’s *τ* coefficient^29^. A value close to 1 means that the administrative units are ordered in the same way, while a value close to 0 means that there is no concordance in the rankings.

### Sensitivity analysis

There are two main sources of uncertainty in the mapping of potential paths for livestock movements: the parameters *δ*_*W*_ and *δ*_*R*_ used to combine the different layers and the weights used to model the land use conductance (Table 1). We used as reference the parameter values *δ*_*W*_ = 0.8 and *δ*_*R*_ = 0.1. This means that the walking layer based on land use information accounts for 80% of the road network importance and the border has a very low permeability (10% of the conductance of the road/walking layer *R*). The reference for the land use weights are displayed in Table 1 according to the season. For both sources of uncertainty, we rely on the Kendall’s *τ* coefficient to compare the ranking of administrative units obtained with the reference distribution of activity with the ones obtained with different parameters and land use weight values. The two sources of uncertainty have been evaluated independently. For the parameters *δ*_*W*_ and *δ*_*R*_, we generated 25 rankings obtained with different pairs of values ranging between 0 and 1 by step of 0.25. For the land use weights, we changed one-at-a-time the weight of the different land use types by adding or subtracting an amount Δ = 0.05 or Δ = 0.1 from the original value.

## Results

### Mobility network analysis

**Figure 4:**
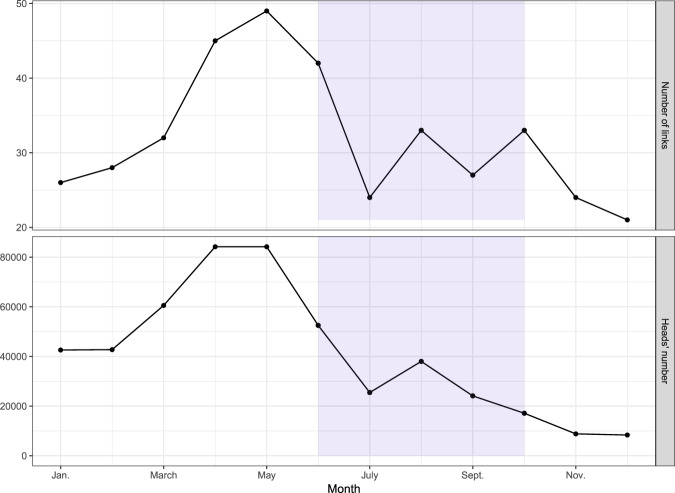
Network variation in 2014. Number of links (top) and number of head displaced (bottom) depending on the month. The shaded area represents the wet season.

Figure 4 shows the changes in the network measured throughout 2014, focusing on the number of links and animals transported each month. As can been seen, most of the activity is concentrated in the months before the wet season (April-June), when the scarcity of rainfall impedes the regeneration of pastures and animals are moved looking for better places. It is worth noting that the wet season (shaded area) is characterized by a dramatic reduction of links and animal movements.

**Figure 5:**
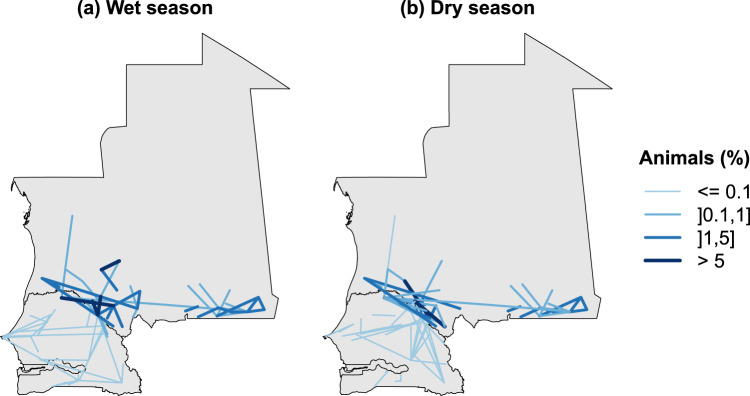
Cattle Mobility Networks in the wet (**a**) and dry (**b**) seasons. The width and the color of a link is proportional to the number of animals displaced. The number of animals displaced from one node to another has been normalized by the total number of animals displaced and is expressed as a percentage.

Table 2 shows the total number of nodes, links and volume of animals displaced depending on the time period. We observe a similar number of links and nodes in the two seasonal networks. We observe however more than twice as many animals are displaced in dry season compared to the wet season. A visual representation of the network in the two seasons is shown in Fig. 5, where link colors and thickness correspond to the number of animals displaced (expressed as a percentage of the total). In both cases, the majority of the links corresponds to movements of small herds and accounted for less than 1% of the total volume. The top 10 links accounted for about 66% of the total volume of animals in the wet season and 75% in the dry season (Table 2). The majority of the animal movements takes place in two areas. The first area is located around the Senegalese-Mauritanian border, with high trade activity between large cities in Mauritania (Nbeika, Boutilimit, Aleg, Mbout, Kaedi and Selibabi) and Senegal (Podor, Matam and Kanel). A major share of these movements involves transboundary movements between Podor and Mbout or between Matam and Kaedi and Mongel, for example. This observation applies to both seasons, but transboundary activity seems to be greater in the wet season than in the dry season. The second area showing major activity is located in southeastern Mauritania close to the border with Mali, involving cities such as Boustaile and Gneiba. It should also be noted that, although more moderate, there is also trade activity between Senegalese cities furthest from the border, such as Kedougou, Diaobe, Tambacounda for the South and Dakar, Diourbel and Touba for the West. That activity is more pronounced during the wet than the dry season.

**Table 2:** Total number of nodes, links and volume of animals according to the season.

Season	Nodes	Links	Volume	Top 10 links volume (%)
All	108	116	0.49	66.83
Wet	85	81	0.16	65.99
Dry	84	78	0.33	74.54

Each node represents an origin or a destination in the livestock mobility network. A link is created between two nodes if at least one animal moves from one node to another. The volume is expressed in million of head.

**Figure 6:**
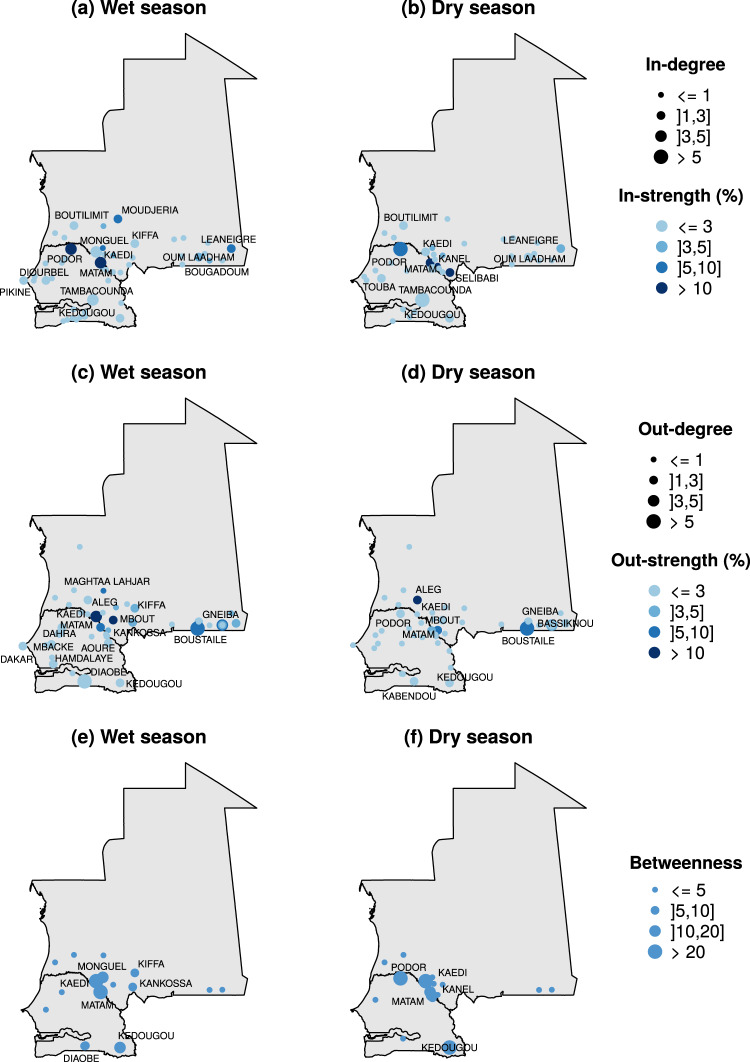
Node centrality analysis. For each node, five centrality indices are displayed for the wet season ((**a**,**c**,**e**)) and the dry season ((**b**,**d**,**f**)): in-degree and in-strength (**a**,**b**), out-degree and out-stength (**c**,**d**) and the betweenness (**e**,**f**). Size of the dots is proportional to the degree (**a**,**d**) or the betweenness (**e**,**f**). Color of the dots corresponds to the in- and out-strength (**a**,**d**). In- and out-strength has been normalized by the total number of animal and are expressed in percentage.

The role played by the different locations slightly changes from one season to another. Figure 6 shows the different locations highlighted according to their centrality. Most of the locations maintain their activity between the two seasons. This is particularly true for the largest market areas of Podor, Kaedi and Matam, located on the border between Senegal and Mauritania, but also for Kedougou in southern Senegal and Boustaile on the border between Mauritania and Mali. They represent major destinations for animal movements. It can be seen in Fig. 6e,f that Podor, on the Senegalese/Mauritanian border is an important transit point during the dry season, but not during the wet season. This network analysis provides useful information about the livestock mobility network in Senegal and Mauritania. However, it does not enable explicit mapping of livestock movements.

### Mapping potential paths for livestock movements

**Figure 7:**
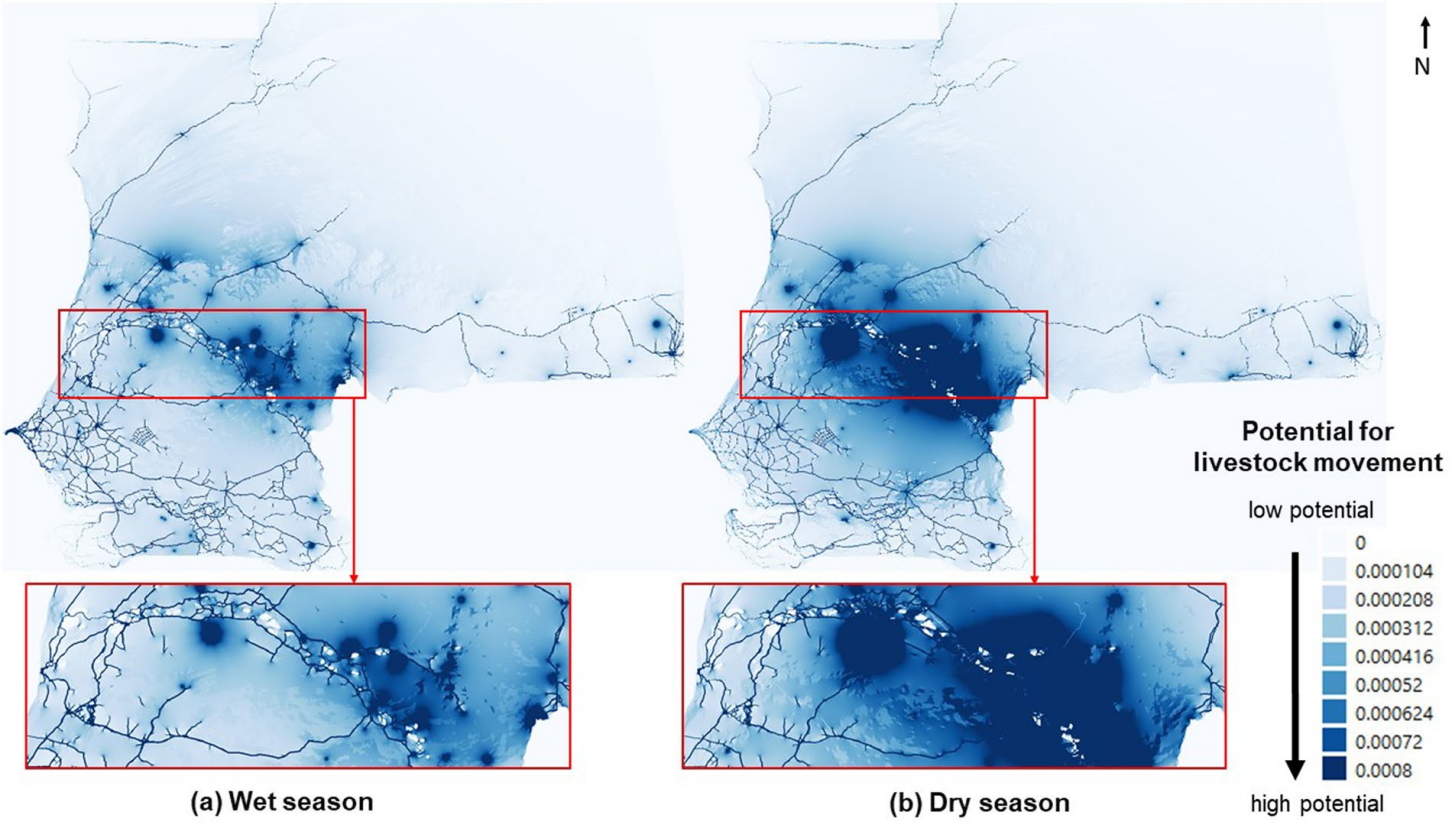
Maps of the potential paths for livestock movements according to the season. (**a**) Wet season. (**b**) Dry season. The maps are based on the parameter values *δ*_*W*_ = 0.8 and *δ*_*R*_ = 0.1 and the land use weights presented in Table 1.

We plotted in Fig. 7 the maps of potential paths for livestock movements in the wet and dry seasons obtained with the landscape connectivity approach. The two maps show different potential movement patterns. For example, the area on the Senegal-Mauritania eastern border is less permeable in the wet season than in the dry season. Moreover, the wet season map shows more complex patterns of passage potential in that area. This was due to the presence of crop plots (see Fig. 3), or floodplains, that animals have to avoid during that season. This highlights the importance of the explicit mapping of network links according to landscape conductance, in order to spatially translate connectivity. For both seasons, the highest potential passages is located around the roads. This is even more pronounced for the wet season, during which some areas could not be crossed and animals are forced to use tracks alongside the roads. Whatever the season, the two maps show one large core area with high crossing potential located on the eastern side of the border between Senegal and Mauritania. Areas located in southern Senegal (Kedougou) and in the southeastern Mauritania (Boustaile) show a low passage potential, while they clearly appear as central nodes in the livestock mobility network (Fig. 6). On the other hand, certain areas located around the Podor-Kaedi-Matam axis exhibit a high passage potential, yet it does not contain any origin or destination nodes. It is typically an area where animals pass through and crossbred, which our methodology enables us identify and delimit. This shows the relevance of landscape connectivity based approaches for identifying areas with a high potential for livestock movements.

### Identification of high potential areas

**Figure 8:**
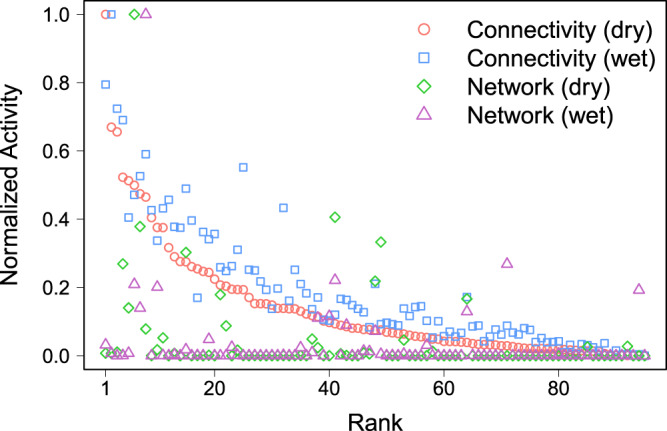
Rank-size distribution of the normalized activity obtained with the landscape connectivity and the network approaches. The total activity (potential livestock movements for the landscape connectivity and total of out- and in-strength for the network approach) contained in each administrative unit have been considered and each distribution have been normalized by its maximum value. The values are ordered according to the activity obtained with the landscape connectivity approach in dry season. Values obtained with the landscape connectivity approach have been calculated with the parameter values *δ*_*W*_ = 0.8 and *δ*_*R*_ = 0.1 and the land use weights presented in Table 1.

**Figure 9:**
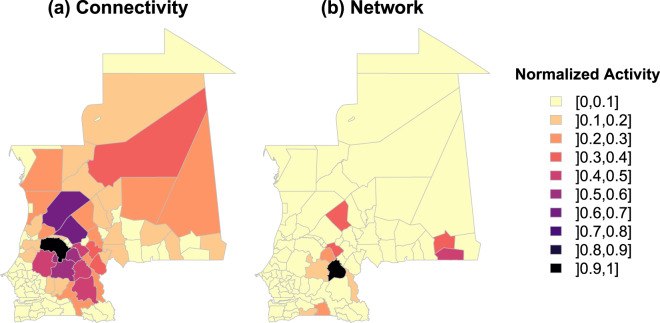
Maps of the normalized activity obtained with our method (**a**) and a network approach (**b**) in the dry season. The total activity (potential livestock movements for the landscape connectivity and total of out- and in-strength for the network approach) contained in each administrative unit have been considered and each distribution have been normalized by its maximum value. Values obtained with the landscape connectivity approach have been calculated with the parameter values *δ*_*W*_ = 0.8 and *δ*_*R*_ = 0.1 and the land use weights presented in Table 1.

We plot in Fig. 8 the rankings of regional administrative units obtained with the different methods (landscape connectivity and network approaches) in the dry and wet seasons. We observe that there was a large difference between administrative unit rankings obtained with the landscape connectivity and network approaches, whatever the season. This is not really surprising, since the two types of activity are not based on the same information, but it highlights the importance of spatially mapping potential paths to identify active areas in terms of animal movements. In particular, there are several units with no activity according to the mobility network that are in the top 10 for the activity measured with the landscape connectivity approach. Maps of the spatial distribution of activity measured with the two approaches in the dry season can be found in Fig. 9. To quantify these differences more rigorously, we computed the correlation between the different rankings with the Kendall’s *τ* coefficient as described in the Material and methods section. Table 3 shows the correlation matrix comparing the four distributions displayed in Fig. 8. We observe a low correlation between connectivity and network approaches whatever the season, thus confirming the results observed in Fig. 8. We also note a strong correlation (*τ* = 0.84) between the rankings obtained with the landscape connectivity approach in wet and dry seasons. It is interesting to note that this correlation falls to 0.66 when comparing the network approach in the wet and dry seasons.

**Table 3:** Kendall rank correlation coefficient matrix.

	Connectivity (dry)	Connectivity (wet)	Network (dry)	Network (wet)
Connectivity (dry)	1	0.84 [0.76, 0.89]	0.4 [0.22, 0.56]	0.29 [0.09, 0.46]
Connectivity (wet)		1	0.41 [0.23, 0.57]	0.31 [0.12, 0.48]
Network (dry)			1	0.66 [0.53, 0.76]

Kendall’s *τ* coefficient between the four rankings displayed in Fig. 8 (Landscape connectivity approach and network approach in dry and wet seasons). Values in bracket correspond to the confidence interval of the correlation coefficient at 95%.

### Results of the sensitivity analysis

**Figure 10:**
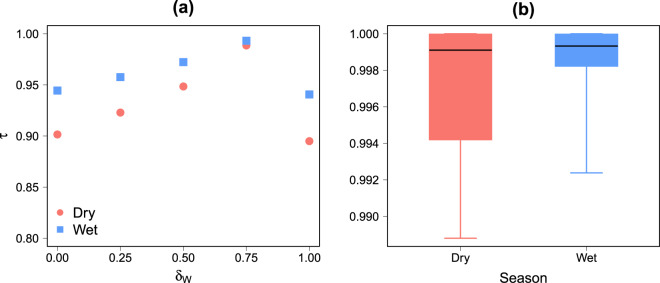
Parameters (**a**) and land use weights (**b**) sensitivity analysis in dry and wet seasons. (**a**) Kendall’s *τ* coefficient between the reference ranking and the ranking obtained with different parameter values as a function of *δ*_*W*_. For each *δ*_*w*_, the *τ* values have been averaged over *δ*_*R*_ values. The value of *τ* for each couple of (*δ*_*W*_, *δ*_*R*_) are available in Supplementary Information (Tables S1 and S2). (**b**) Boxplots of the Kendall’s *τ* coefficient between the reference ranking and the ranking obtained with different land use weight values. Each boxplot is composed of the minimum, the lower hinge, the median, the upper hinge and the maximum. The value of *τ* for each land use weight values are available in Supplementary Information (Tables S3 and S4).

Figure 10 shows the results of the parameters and land use weights sensitivity analysis in the dry and wet seasons. We observe in Fig. 10a that the similarity between the ranking of reference and the ones obtained with different *δ*_*W*_ values is globally high with a Kendall’s *τ* coefficient ranging from 0.8 to 1. The similarity decreases slowly when *δ*_*W*_ decreases below the reference value, we observe a break of this trend when *δ*_*W*_ = 1. In this particular case, the results are no longer driven by the road network, leading to a modification in the potential movement patterns on a global scale. Note that since *δ*_*R*_ has almost no impact on the activity at a global scale (see Table S1 and S2 in Supplementary Information for more details), for each *δ*_*w*_ value, the *τ* values have been averaged over *δ*_*R*_. It is however important to keep in mind that the effect of *δ*_*R*_ is probably higher at a local scale since it only affected areas close to the Mauritanian-Senegalese border. As can be observed in Fig. 10b changes in land use weight values have very little impact on the rankings (see Table S3 and S4 in Supplementary Information for more details). In both cases, the sensitivity of the results to variations in parameters and land use weight values is higher in the dry than in the wet seasons.

## Discussion

The precise description of livestock movement patterns has a central role in many applied questions. This is particularly true in Sahelian semi-arid regions, where it has become a crucial requirement to help decision-makers in dealing with conflicts between herders and farmers, or regarding the spread of animal diseases. The originality of the approach proposed in this article lies in the fine mapping of animal flows by weighting a conductance map by the number of head of livestock. The resulting raster map reflects the potential for livestock movement in each pixel according to its landscape connectivity and its position relative to the livestock mobility network. We illustrated our approach with a livestock mobility network in Senegal and Mauritania in the 2014 dry and wet seasons, which we combined with different land-use information (land cover, roads and borders). Our results demonstrate the robustness of our approach in identifying and ranking areas according to their potential for livestock movement. Other applications from our methodology are now possible. For example, we could cross the information contained in our potential maps with risk factors for the spread of diseases like Rift Valley fever^7^. It will conduct to identify areas with the highest risk of disease transmission. When crossing the maps stemming from the landscape connectivity approach with maps of cropped areas, we can also identify priority zones where passage corridors have to be settled and secured, as these zones have the highest risk of conflicts between farmers and breeders.

### Limitations of the study

It needs to be kept in mind that our approach is highly dependent on the data being used and their resolution. The resolution of the conductance map, at 500 meters in our study, depends on the resolution of the land cover map and might not enable the consideration of very fine paths. Our results showed that the potential map was mostly driven by the road network, which can also be a major source of uncertainty.

Many factors drive mobility dynamics: landscape configuration, road quality, need for food, need for watering points, border crossing, religious feasts, etc. The conductance map has to include all these mobility-driven factors. For this study, we were able to collect most of the geographical layers for each of these factors, except that of the watering points (boreholes and ponds). Consequently, the maps obtained in this study do not take into consideration the need to pass through watering points, especially during the dry season. This is an important drawback counterbalanced by the fact that Senegal and Mauritania have a very dense grid of boreholes.

Another difficulty is the reliability of the mobility data. Mobility data were collected using two different approaches in Senegal and Mauritania. For the Mauritanian case, a synthetic survey was conducted by the National Livestock and Veterinary Research Centre (CNERV) and compared with health certificates collected by Veterinarian Offices. In the case of Senegal, paper copies of sanitary movement permits (LPS) were collected by ad-hoc activities. These certificates provided information about origins and destinations, and we do not know if the composition of the herd changed during the journey due to animal sales. Furthermore, there was no proof that the herds actually reached their destination. Another bias in the data was linked to the fact that this data set did not include undeclared movements (for herds that did not have a sanitary movement permit).

Lastly, construction of the conductance map, which is the basis of the proposed methodology, relies on resistance weights given by experts. It should be noted that the main purpose of this article was to propose a methodology and we did not try to increase the number of experts. Nevertheless, we showed that small variations applied one at a time to the land use weight values have no significant effect on the rankings. To use the presented method for operational purposes, concerted thought needs to be given to the weights to be assigned, and a multivariate sensitivity analysis of these weights needs to be integrated into the approach.

### Concluding remarks

The identification of high potential for livestock movements is a core issue for decision-makers, whether in the field of animal health or territorial planning. Our approach opens up some interesting perspectives for modeling potential animal passage in semi-arid regions experiencing a lack of specific data on livestock movements. It is, however, important to note that a large share of livestock remains in its zone of origin. These sedentary animals are often in contact with transhumant animals that cross their territory. This information should be added, to complete the map of the potential for livestock movements provided in this study.

## Supplementary information

Supplementary information.

## References

[1] Corniaux C (2014). Le commerce du bétail sahélien. Une filière archaïque ou la garantie d’un avenir prometteur?. Afrique contemporaine.

[2] Kamuanga, M. J. B., Somda, J., Sanon, Y. & Kagoné, H. Livestock and regional market in the Sahel and West Africa. *Tech. Rep*. (2008).

[3] Corniaux, C., Ancey, V., Touré, I., Diao Camara, A. & Cesaro, J.-D. Pastoral mobility, from a Sahelian to a sub-regional issue, 60–61 (Cirad, Nepad, Montpellier, 2016).

[4] Moritz M (2006). Changing Contexts and Dynamics of Farmer-Herder Conflicts across West Africa. Can. J. Afr. Stud..

[5] Turner MD, Ayantunde AA, Patterson KP, Patterson ED (2011). Livelihood Transitions and the Changing Nature of Farmer–Herder Conflict in Sahelian West Africa. The. J. Dev. Stud..

[6] Turner MD (2011). The New Pastoral Development Paradigm: Engaging the Realities of Property Institutions and Livestock Mobility in Dryland Africa. Soc. & Nat. Resour..

[7] Tran A (2016). Development and Assessment of a Geographic Knowledge-Based Model for Mapping Suitable Areas for Rift Valley Fever Transmission in Eastern Africa. PLOS Neglected Trop. Dis..

[8] Napp S (2018). Understanding the legal trade of cattle and camels and the derived risk of Rift Valley Fever introduction into and transmission within Egypt. PLOS Neglected Trop. Dis..

[9] Fournié G (2018). A dynamic model of transmission and elimination of peste des petits ruminants in Ethiopia. Proc. Natl. Acad. Sci..

[10] Volkova VV, Howey R, Savill NJ, Woolhouse MEJ (2010). Sheep Movement Networks and the Transmission of Infectious Diseases. PLoS ONE.

[11] Bajardi P, Barrat A, Savini L, Colizza V (2012). Optimizing surveillance for livestock disease spreading through animal movements. J. The Royal Soc. Interface.

[12] Hardstaff, J. L., Hüsler, B. & Rushton, J. R. Livestock trade networks for guiding animal health surveillance. *BMC Vet. Res*. **11** (2015). 10.1186/s12917-015-0354-4 PMC4411738 25889738

[13] Dean AS (2013). Potential Risk of Regional Disease Spread in West Africa through Cross-Border Cattle Trade. PLoS ONE.

[14] Motta, P. *et al*. Implications of the cattle trade network in Cameroon for regional disease prevention and control. *Sci. Reports* **7** (2017). 10.1038/srep43932 PMC5339720 28266589

[15] Nicolas G (2018). Predictive gravity models of livestock mobility in Mauritania: The effects of supply, demand and cultural factors. PLOS ONE.

[16] Apolloni A (2018). Towards the description of livestock mobility in Sahelian Africa: Some results from a survey in Mauritania. PLOS ONE.

[17] Mekonnen, G. A., Ameni, G., Wood, J. L. N., Berg, S. & Conlan, A. J. K. Network analysis of dairy cattle movement and associations with bovine tuberculosis spread and control in emerging dairy belts of Ethiopia. *BMC Vet. Res*. **15** (2019). 10.1186/s12917-019-1962-1 PMC6660945 31349832

[18] Chaters GL (2019). Analysing livestock network data for infectious disease control: an argument for routine data collection in emerging economies. Philos. Transactions Royal Soc. B: Biol. Sci..

[19] Guo D, Zhu X, Jin H, Gao P, Andris C (2012). Discovering Spatial Patterns in Origin-Destination Mobility Data: Discovering Spatial Patterns in Origin-Destination Mobility Data. Transactions GIS.

[20] Adriansen HK, Nielsen TT (2005). The geography of pastoral mobility: A spatio-temporal analysis of GPS data from Sahelian Senegal. GeoJournal.

[21] Motta, P. *et al*. Cattle transhumance and agropastoral nomadic herding practices in Central Cameroon. *BMC Vet. Res*. **14** (2018). 10.1186/s12917-018-1515-z PMC6029425 29970084

[22] Aubauge, S. *et al*. Pastoral livestock farming in Sahel and West Africa (2017).

[23] McRae BH, Beier P (2007). Circuit theory predicts gene flow in plant and animal populations. Proc. Natl. Acad. Sci..

[24] Brodie JF (2015). Evaluating multispecies landscape connectivity in a threatened tropical mammal community. Conserv. Biol..

[25] Mateo-Sanchez MC, Balkenhol SN, Cushman PT, Dominguez A, Saura S (2015). Estimating effective landscape distances and movement corridors: Comparison of habitat and genetic data. Ecosphere..

[26] Bishop-Taylor R, Tulbure MG, Broich M (2015). Surface water network structure, landscape resistance to movement and flooding vital for maintaining ecological connectivity across Australia’s largest river basin. Landsc. Ecol..

[27] McClure ML, Hansen A, Inman RM (2016). Connecting models to movements: testing connectivity model predictions against empirical migration and dispersal data. Landsc. Ecol..

[28] Tatem A, Hemelaar J, Gray RR, Salemi M (2012). Spatial accessibility and the spread of HIV-1 subtypes and recombinants. AIDS.

[29] Puka, L. Kendall’s Tau, 713–715 (Springer Berlin Heidelberg, Berlin, Heidelberg, 2011).

