# Supplementary material for: Mapping livestock movements in Sahelian Africa
Source: Sci Rep. 2020 May 20;10:8339. doi: 10.1038/s41598-020-65132-8 (PMC7239863; doi:10.1038/s41598-020-65132-8)
Supplement: Supplementary file 1 — Supplementary information. [file 41598_2020_65132_MOESM1_ESM.pdf]

Supplementary Information for Mapping livestock movements in Sahelian Africa

Camille Jahel[1,2,3,*+], Maxime Lenormand[4,*+], Ismaila Seck[5,6], Andrea Apolloni[7,8], Ibra Toure[9], Coumba Faye[5], Baba Sall[5], Mbargou Lo[5], Cécile Squarzoni Diaw[7], Renaud Lancelot[7], and Caroline Coste[7]

[1]CIRAD, UMR TETIS, 500 rue JF Breton, FR-34093 Montpellier, France
[2]ISRA, BAME, rue des Hydrocarbure, Dakar, Senegal
[3]PPZS, Pastoral Systems and Dry Lands, Dakar, Senegal
[4]TETIS, Univ Montpellier, AgroParisTech, Cirad, CNRS, INRAE, Montpellier, France
[5]DSV, Dakar, Senegal
[6]FAO, Regional Office
[7]CIRAD, UMR ASTRE, Campus International de Baillarguet, 34398 Montpellier, France
[8]ISRA, LNERV, rue fond de terre, Dakar, Senegal
[9]CIRAD, UMR SELMET, Campus International de Baillarguet, 34398 Montpellier, France
[*]Corresponding authors: camille.jahel@cirad.fr & maxime.lenormand@inrae.fr
[+]These authors contributed equally to this work

ABSTRACT

In the dominant livestock systems of Sahelian countries herds have to move across territories. Their mobility is often a source of conflict with farmers in the areas crossed, and helps spread diseases such as Rift Valley Fever. Knowledge of the routes followed by herds is therefore core to guiding the implementation of preventive and control measures for transboundary animal diseases, land use planning and conflict management. However, the lack of quantitative data on livestock movements, together with the high temporal and spatial variability of herd movements, has so far hampered the production of fine resolution maps of animal movements. This paper proposes a general framework for mapping potential paths for livestock movements and identifying areas of high animal passage potential for those movements. The method consists in combining the information contained in livestock mobility networks with landscape connectivity, based on different mobility conductance layers. We illustrate our approach with a livestock mobility network in Senegal and Mauritania in the 2014 dry and wet seasons.

Supplementary Figures

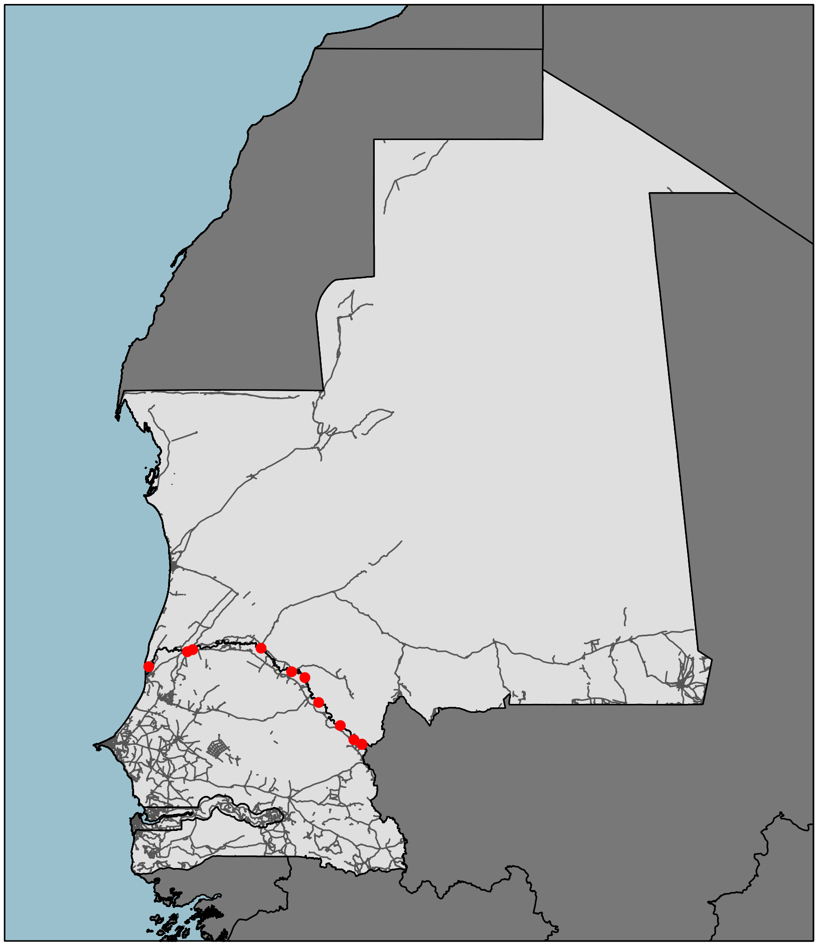

Figure S1. Senegal, Gambia and Mauritania's main road network. The red points represent the border checkpoints. The main road network in Senegal, Gambia and Mauritania has been downloaded from OpenStreetMap (data available online at https://www.openstreetmap.org, last accessed 18/02/2020). OpenStreetMap is made available under the Open Database License http://opendatacommons.org/licenses/odbl/1.0/. Any rights in individual contents of the database are licensed under the Database Contents License: http://opendatacommons.org/licenses/dbcl/1.0/.

Supplementary Tables

	$\delta_R = 0$	$\delta_R = 0.25$	$\delta_R = 0.5$	$\delta_R = 0.75$	$\delta_R = 1$
$\delta_W = 0$	0.9015	0.9015	0.9015	0.9028	0.9006
$\delta_W = 0.25$	0.9230	0.9230	0.9230	0.9230	0.9230
$\delta_W = 0.5$	0.9489	0.9489	0.9489	0.9485	0.9471
$\delta_W = 0.75$	0.9892	0.9892	0.9892	0.9897	0.9857
$\delta_W = 1$	0.8952	0.8952	0.8952	0.8952	0.8943

Table S1. Parameter sensitivity analysis in dry season. Kendall's τ coefficient between the ranking obtained with the reference distribution ($\delta_W = 0.8$ and $\delta_R = 0.1$) and the rankings obtained with different couples (δ_W,δ_R) values ranging between 0 and 1 in dry season. In all cases the conductance maps have been computed with the land use weights in dry season as defined in Table 1 in the main text.

	$\delta_R = 0$	$\delta_R = 0.25$	$\delta_R = 0.5$	$\delta_R = 0.75$	$\delta_R = 1$
$\delta_W = 0$	0.9449	0.9449	0.9449	0.9454	0.9422
$\delta_W = 0.25$	0.9579	0.9579	0.9579	0.9579	0.9566
$\delta_W = 0.5$	0.9727	0.9727	0.9727	0.9727	0.9709
$\delta_W = 0.75$	0.9942	0.9942	0.9937	0.9937	0.9901
$\delta_W = 1$	0.9409	0.9409	0.9404	0.9409	0.9404

Table S2. Parameter sensitivity analysis in wet season. Kendall's τ coefficient between the ranking obtained with the reference distribution ($\delta_W = 0.8$ and $\delta_R = 0.1$) and the rankings obtained with different couples (δ_W,δ_R) values ranging between 0 and 1 in wet season. In all cases the conductance maps have been computed with the land use weights in wet season as defined in Table 1 in the main text.

Land use type	$\Delta = -0.1$	$\Delta = -0.05$	$\Delta = 0.05$	$\Delta = 1$
Coastal strip	1	1	1	1
Mangrove	1	1	0.9996	0.9991
Water bodies	0.9991	0.9996	0.9996	0.9991
Irrigated croplands	-	-	-	-
Croplands	0.9892	0.9933	-	-
Forest area	0.9987	0.9991	0.9987	0.9973
Mosaic croplands & grassland	0.9888	0.9937	-	-
Open grassland	0.9892	0.9928	-	-
Dune and peneplain pastures	0.9928	0.9955	0.9942	0.9892
Dune and gravel pastures	0.9897	0.9946	-	-
Salt land	0.9996	1	-	-
Bare rock	0.9982	0.9991	0.9987	0.9982
Urban area	1	1	1	1
Major rivers	-	-	-	-

Table S3. Land use weight sensitivity analysis in dry season. Kendall's τ coefficient between the ranking obtained with the reference distribution (land use weight in dry season as defined Table 1 in the main text) and the rankings obtained with small variation Δ applied on the original values ranging between -0.1 and 0.1 when applicable. In all cases the maps of potential paths have been obtained with the parameters $\delta_W = 0.8$ and $\delta_R = 0.1$.

Land use type	$\Delta = -0.1$	$\Delta = -0.05$	$\Delta = 0.05$	$\Delta = 1$
Coastal strip	0.9996	0.9996	1	1
Mangrove	1	1	1	1
Water bodies	1	1	1	1
Irrigated croplands	-	-	-	-
Croplands	0.9987	0.9996	0.9982	0.9955
Forest area	0.9982	0.9987	0.9991	0.9987
Mosaic croplands & grassland	0.9996	0.9996	0.9978	0.9964
Open grassland	0.9937	0.9960	-	-
Dune and peneplain pastures	0.9969	0.9987	0.9969	0.9924
Dune and gravel pastures	0.9942	0.9969	0.9982	0.9951
Salt land	0.9991	0.9996	1	0.9996
Bare rock	0.9982	0.9991	0.9996	0.9987
Urban area	1	1	1	1
Major rivers	-	-	-	-

Table S4. Land use weight sensitivity analysis in wet season. Kendall's τ coefficient between the ranking obtained with the reference distribution (land use weight in wet season as defined Table 1 in the main text) and the rankings obtained with small variation Δ applied on the original values ranging between -0.1 and 0.1 when applicable. In all cases the maps of potential paths have been obtained with the parameters $\delta_W = 0.8$ and $\delta_R = 0.1$.
